# Monetary Incentives Modulate Feedback-related Brain Activity

**DOI:** 10.1038/s41598-018-30294-z

**Published:** 2018-08-09

**Authors:** Shuting Mei, Qi Li, Xun Liu, Ya Zheng

**Affiliations:** 1 Department of Psychology, Dalian Medical University, Dalian, China; 2 CAS Key Laboratory of Behavioral Science, Institute of Psychology, Beijing, China; 3 Department of Psychology, University of Chinese Academy of Sciences, Beijing, China

## Abstract

Previous research has shown that feedback evaluation is sensitive to monetary incentive. We investigated whether this sensitivity is driven by motivational salience (the difference between both rewarding and punishing events versus neutral events) or by motivational valence (the difference between rewarding and punishing events). Fifty-seven participants performed a monetary incentive delay task under a gain context, a loss context, and a neutral context with their electroencephalogram recorded. During the time domain, the feedback-related negativity (FRN) showed a motivational salience effect whereas the P3 displayed a reward valence effect. During the time-frequency domain, we observed a motivational salience effect for phase-locked theta power regardless of performance feedback, but a reward valence effect for non-phase-locked theta power in response to unsuccessful feedback. Moreover, we found a reward valence effect for phase-locked delta. These findings thus suggest that the affective modulation on feedback evaluation can be driven either by motivational valence or by motivational salience, which depends on the temporal dynamics (the FRN vs. the P3), the frequency dynamics (theta vs. delta power), as well as the phase dynamics (evoked vs. induced power).

## Introduction

The ability to evaluate the consequences of one’s own actions is critical for adaptive behavior such that a positive outcome makes an action more likely to be repeated, while a negative outcome makes the action less likely to be continued^1^. In addition to its performance-signaling function, a feedback stimulus can deliver motivational information. Specifically, a feedback stimulus in a reward context indicates whether or not a response should be rewarded and thus serves a reward-signaling function, whereas in a punishment context it indicates whether or not a response should be punished and thus serves a punishment-signaling function. Although reward and punishment have opposite motivational valence (a positive valence for reward and a negative connotation for punishment), they convey similar motivational salience, a quantity that is high for both rewarding and punishing events and is low for motivationally neutral events^2^. Thus, the question arises whether feedback evaluation is modulated by motivational salience (the difference between both rewarding and punishing events versus neutral events) or by motivational valence (the difference between rewarding and punishing events, including reward valence reflected as reward > punishment and punishment valence reflected as punishment > reward).

Event-related potential (ERP) studies of feedback processing have focused on the feedback-related negativity (FRN). The FRN is a frontocentral negative deflection that occurs between 200 and 350 ms following the onset of a feedback stimulus and appears to be generated in the anterior midcingulate cortex (aMCC)^3^ as well as the striatum^4^. The FRN is typically observed under uncertain task contexts in which participants try to learn optimal response patterns, such as learning tasks^3,5^ or gambling tasks^6^, and is usually more negative for unfavorable compared to favorable feedback. The reinforcement learning theory proposes that the FRN reflects the impact of the phasic signal of mesencephalic dopamine system on the aMCC^5^. Phasic decreases in dopamine activity elicited by negative feedback disinhibit motor neurons in the aMCC and thereby increase the amplitude of the FRN^7^. By contrast, phasic increases in dopamine activity elicited by positive feedback inhibit motor neurons in the aMCC and thus suppress the amplitude of the FRN^8^. However, other theories highlight the influence of affect and motivation significance on the FRN amplitude^6,9^. More recently, the activity in the time window of the FRN has been interpreted as that a reward positivity selectively elicited by rewards is superimposed on a baseline negativity (i.e., the N2 component) elicited by both rewards and losses^10–13^.

Despite numerous studies associating the FRN either with the performance-signaling function or with the affect/motivation-signaling function, the interface between feedback evaluation and motivational significance is still unclear. Specifically, most previous studies confounded performance feedback with motivational feedback in that a monetary gain feedback indicates both a correct performance and a gain associated with the correct performance, whereas a monetary loss feedback indicates both an incorrect performance and a loss incurred by the incorrect performance^6,14–22^. One approach to disentangling the effects of reward and punishment on feedback evaluation is to include a neutral condition during which only correct and incorrect performance feedback are delivered and then to compare the gain and loss conditions with the neutral condition.

Several previous studies have demonstrated that electrophysiological activity during feedback evaluation can be enhanced by monetary rewards by comparing the FRN elicited in a reward condition during which participants received feedback indicating that they either performed correctly and won or performed incorrectly and failed to win money with the FRN elicited in a neutral condition during which they received feedback indicating that they performed either correctly or incorrectly^23–25^. However, one limitation of these ERP studies is that only a gain condition and a neutral condition were included, thus making it unclear whether the modulation of feedback evaluation by monetary incentive reflects a reward system, or in a more general term, a motivational/arousing system.

To our knowledge, only three studies have attempted to address this issue by including a gain condition, a loss condition, and a neutral condition in a monetary incentive delay (MID) task, but reported inconsistent results^26–28^. Broyd *et al*. only compared FRNs elicited by negative feedback during the three conditions and found that negative feedback in the neutral condition elicited a comparable FRN relative to the gain condition, but a decreased FRN compared to the loss condition, indicating an enhanced punishment sensitivity during feedback evaluation^26^. In contrast, Gu *et al*. observed a motivational salience effect, regardless of performance feedback, such that a larger FRN was elicited during the neutral condition compared to both the gain and loss conditions, with no significant difference between the latter two conditions^27^. Finally, Pfabigan *et al*. found that the modulation of monetary incentive on feedback evaluation was dependent on performance feedback. Specifically, the FRN in response to positive feedback was increased for the neutral condition compared to the loss condition, which elicited in turn a larger FRN than the gain condition. In contrast, the FRN in response to negative feedback was increased for the neutral condition versus the gain condition, which elicited in turn an enhanced FRN versus the loss condition. These inconsistences may result from methodological factors among these studies. For example, both Broyd *et al*. and Gu *et al*. used a higher success probability (~66%), leading to an asymmetric outcome ratio. While this confounding variable was controlled by Pfabigan *et al*., the visual characteristics of feedback stimuli in this study were different across conditions. Given that the FRN is susceptible to outcome probability and visual characteristic^29–32^, well controlled tasks are needed to address this question.

Besides these methodological issues, another main problem of the FRN component concerns component overlap such that it is often distorted by the preceding P2 component and the following P3 component^33^. A complementary method that partially circumvents this problem is to decompose the neural signals during the FRN time window into separate frequency bands. Recent studies using time-frequency decomposition methods have demonstrated that the neural signals during the FRN time window consist mainly of two oscillations: theta and delta power^34,35^. Several research has demonstrated that whereas theta activity is more sensitive to negative (loss and incorrect) feedback, delta activity is more sensitive to positive (gain and correct) feedback^34–38^. Using a reward time-estimation task, Pornpattananangkul and Nusslock reported that both theta and delta power, regardless of performance feedback, were increased under a gain condition compared to a neutral condition, thus reflecting a sensitivity to reward processing^37^. This study, however, had not included a loss condition.

Here, we addressed the question whether feedback evaluation is modulated by motivational valence specifically or motivational salience generally. To this end, participants performed a modified version of the MID task^39^ with successful versus unsuccessful performance feedback delivered under a gain context, a loss context, and a neutral context. Using a staircase algorithm, the success rate during each context was approximately 50%. Moreover, performance feedback across contexts was indicated by the same stimulus, making condition comparisons more reasonable. We collected the electroencephalogram (EEG) signals of feedback processing from both the time domain (i.e., the FRN and P3) and the time-frequency domain (i.e., delta and theta power). If feedback evaluation is specifically more sensitive to motivational valence, these electrophysiological responses would be enhanced in the gain context compared to both the loss and neutral contexts, or in the loss context compared to both the gain and neutral contexts. If feedback evaluation is otherwise more sensitive to motivational salience, these neural signals would be enhanced in both the gain and loss contexts compared to the neutral context, with no significant difference between the gain and loss contexts.

## Results

### Behavioral and rating data

As described in Zhang *et al*.^40^, response times were slowest for the neutral context, intermediate for the loss context, and fastest for the gain context (*p*s < 0.05). Since the staircase algorithm took care of the 50% distribution of the successful and unsuccessful feedback, we also measured the response variance (i.e., the *SD* of response times across trials) for each context. The ANOVA revealed a significant main effect of context, *F*(2, 112) = 5.63, *p* = 0.006. Post hoc comparisons revealed that the response variance in the neutral context (64.96) was significantly larger than in the gain (52.02, *p* = 0.014) and loss (52.42, *p* = 0.039) contexts, with no difference between the latter two contexts (*p* > 0.9). In addition, for successful performance feedback, both valence- and arousal-rating scores were higher for the gain trials than the loss trials, which were in turn higher than the neutral trials (*p*s < 0.005). In contrast, for unsuccessful performance feedback, valence-rating scores were lower for the loss trials than the gain trials, which were in turn lower than the neutral trials (*p*s < 0.05), whereas arousal-rating scores were enhanced for the loss relative to neutral trials (*p* = 0.018). In sum, both behavioral and rating data indicated a response speeding and greater affective experiences for the motivational versus neutral context in the MID task.

### Electrophysiological data

#### Time domain

**Figure 1:**
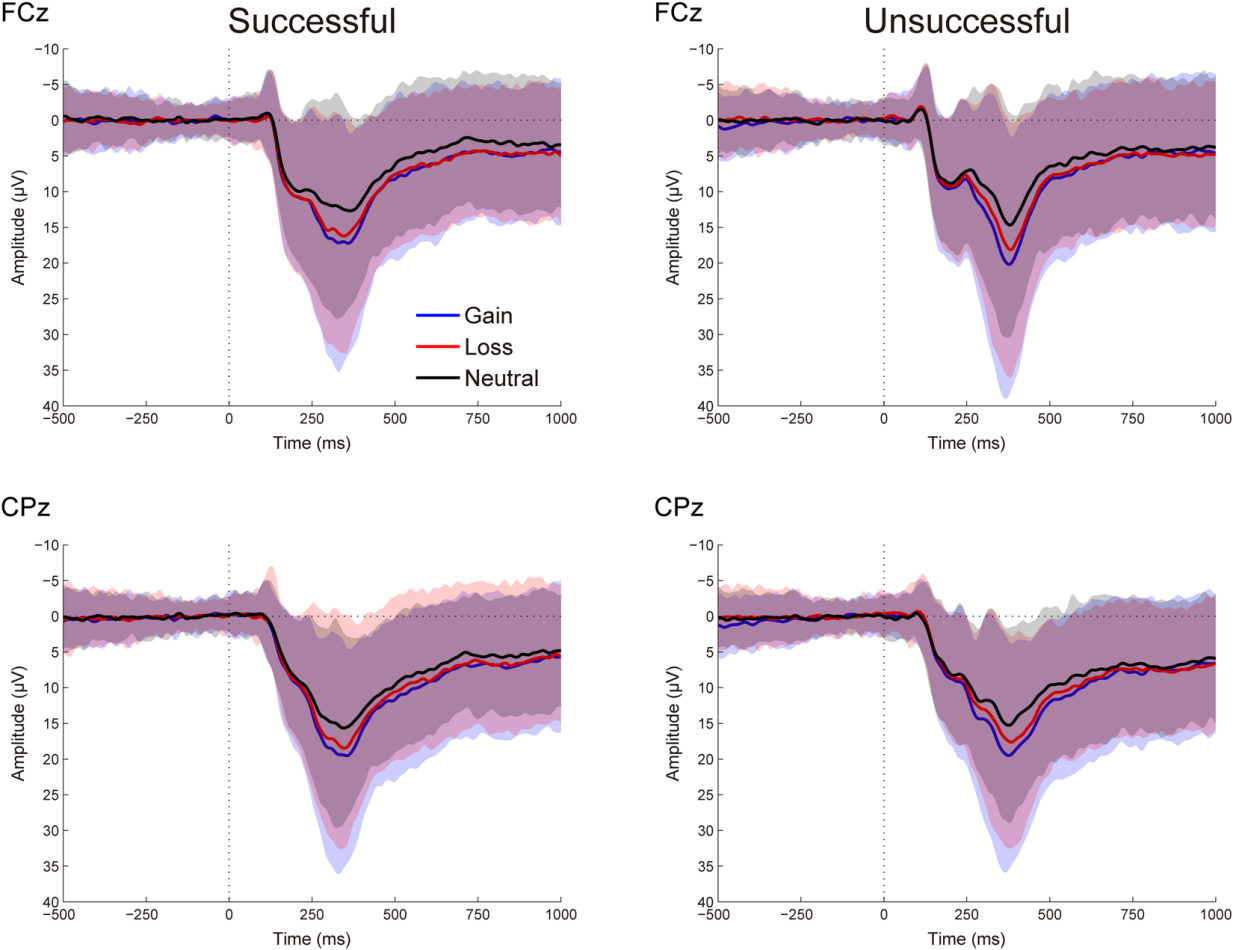
Grand-averaged ERP waveforms in response to successful and unsuccessful performance feedback as a function of context at FCz (top) and CPz (bottom). Shaded areas indicate the confidence intervals of 95% around the means.

**Figure 2:**
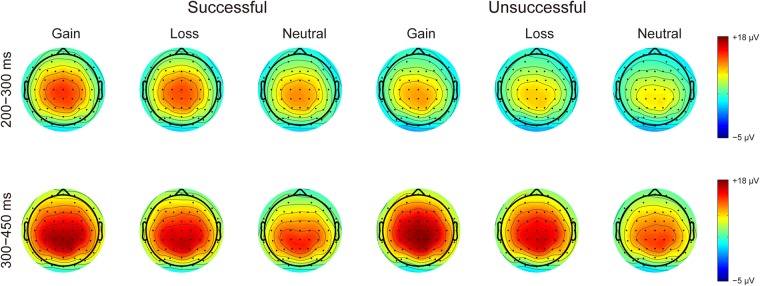
Topographic distribution maps for the FRN (200–300 ms) and P3 (300–450 ms) in response to successful and unsuccessful performance feedback as a function of context.

**Figure 3:**
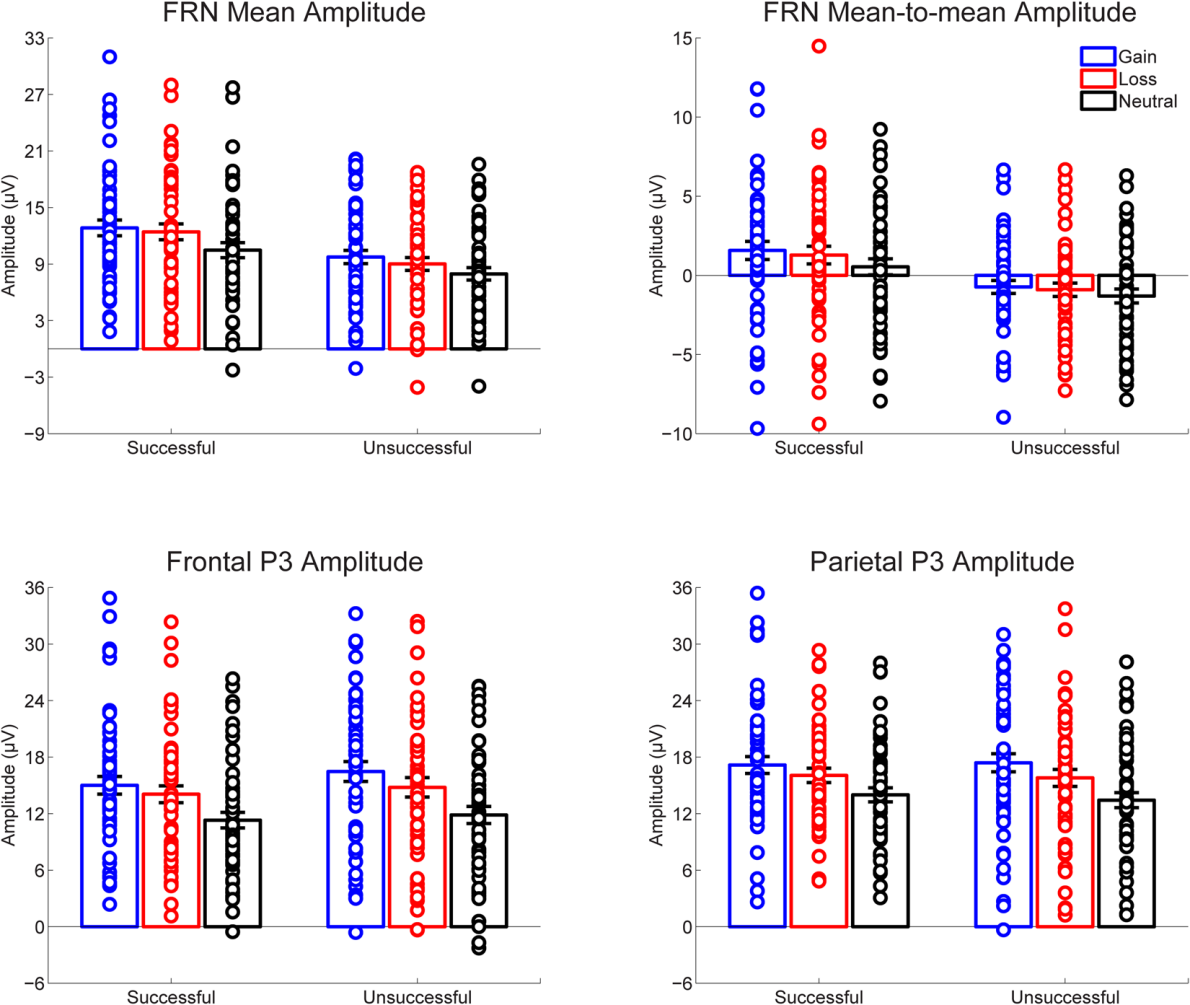
Stripcharts of amplitude data for the FRN and P3 in response to successful and unsuccessful performance feedback as a function of context. Error bars represent standard error of the means.

Figure 1 presents the grand-averaged ERP waveforms elicited by performance feedback as a function of context. The topographic maps for the FRN (200–300 ms) and P3 (300–450 ms) are shown in Fig. 2. The amplitude data of the two components are depicted in Fig. 3. Table 1 summarizes the ANOVA results of the two FRN measures. As expected, feedback indicating unsuccessful performance elicited a larger FRN compared to that indicating successful performance, as revealed by significant main effects of performance for all two FRN measures. Context had a significant effect on the FRN for all two measures. Post hoc comparisons revealed that the FRN was larger for the neutral trials compared to both the gain and loss trials (*p*s < 0.01), with no significant difference between the gain and loss trials (*p*s > 0.09). Moreover, interactions between performance and context failed to achieve significance across the two approaches.

**Table 1:** Summary of the analysis of variance performed on FRN amplitudes with the mean-amplitude approach and the mean-to-mean-amplitude approach.

	Mean amplitude				Mean-to-mean amplitude			
	*df*	*F*	*p*	ω^2^	*df*	*F*	*p*	ω^2^
P	(1,56)	42.26	<0.001	0.252	(1,56)	26.15	<0.001	0.198
C	(2,112)	28.37	<0.001	0.085	(2,112)	7.46	0.001	0.018
P × C	(2,112)	1.69	0.191	0.002	(2,112)	0.60	0.539	<0.001

*Note*. P = Performance; C = Context.

Table 2 summarizes the ANOVA results of the posterior P3 and the anterior P3. The posterior P3 was comparable for successful and unsuccessful performance feedback. However, it varied as a function of context. Post hoc comparisons revealed that the posterior P3 was larger for the gain trials than for both the loss and neutral trials (*p*s < 0.001). In addition, the posterior P3 was enhanced for the loss compared to neutral trials (*p* < 0.001). Similar context effects were observed for the anterior P3, as revealed by a linearly increased P3 from the neutral trials, to the loss trials, and to the gain trials (*p*s < 0.001). Moreover, the anterior P3 was enhanced for unsuccessful compared to successful performance feedback.

**Table 2:** Summary of the analysis of variance performed on posterior (CPz) and anterior (FCz) P3 amplitudes.

	Posterior P3				Anterior P3			
	*df*	*F*	*p*	ω^2^	*df*	*F*	*p*	ω^2^
P	(1,56)	0.27	0.608	<0.001	(1,56)	4.98	0.030	0.016
C	(2,112)	37.59	<0.001	0.241	(2,112)	49.10	<0.001	0.278
P × C	(2,112)	1.25	0.292	0.001	(2,112)	1.48	0.233	0.001

*Note*. P = Performance; C = Context.

#### Time-frequency domain

**Figure 4:**
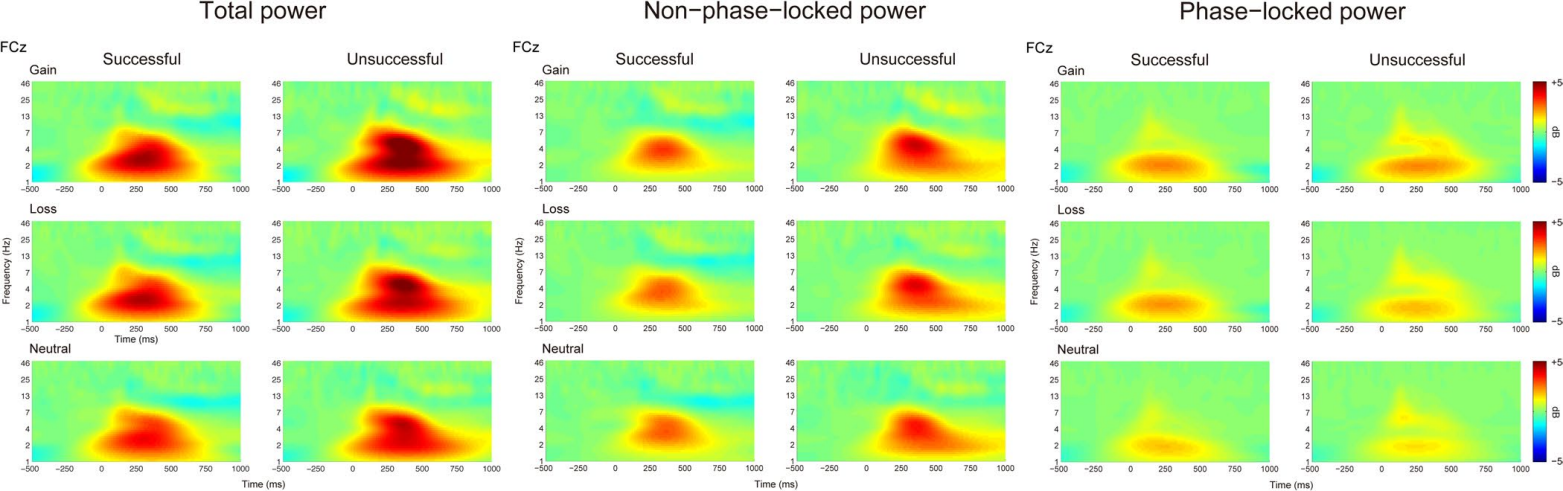
Time-frequency representations of total (left), non-phase-locked (middle) and phase-locked (right) EEG power in response to successful and unsuccessful performance feedback in each context at FCz.

**Figure 5:**
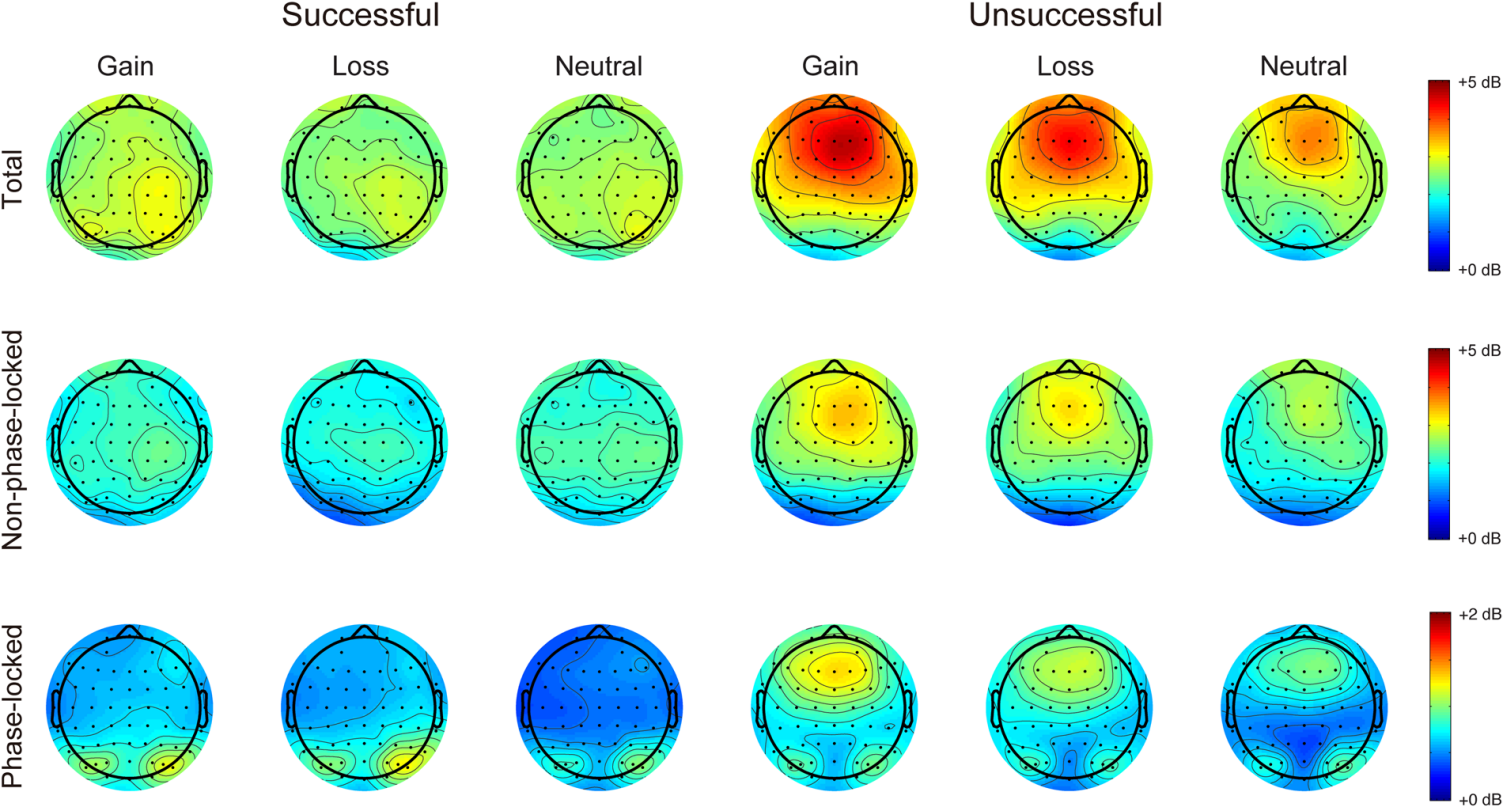
Topographic distribution maps for total, non-phase-lock, and phase-lock theta power (4–6 Hz, 200–500 ms) in response to successful and unsuccessful performance feedback as a function of context.

**Figure 6:**
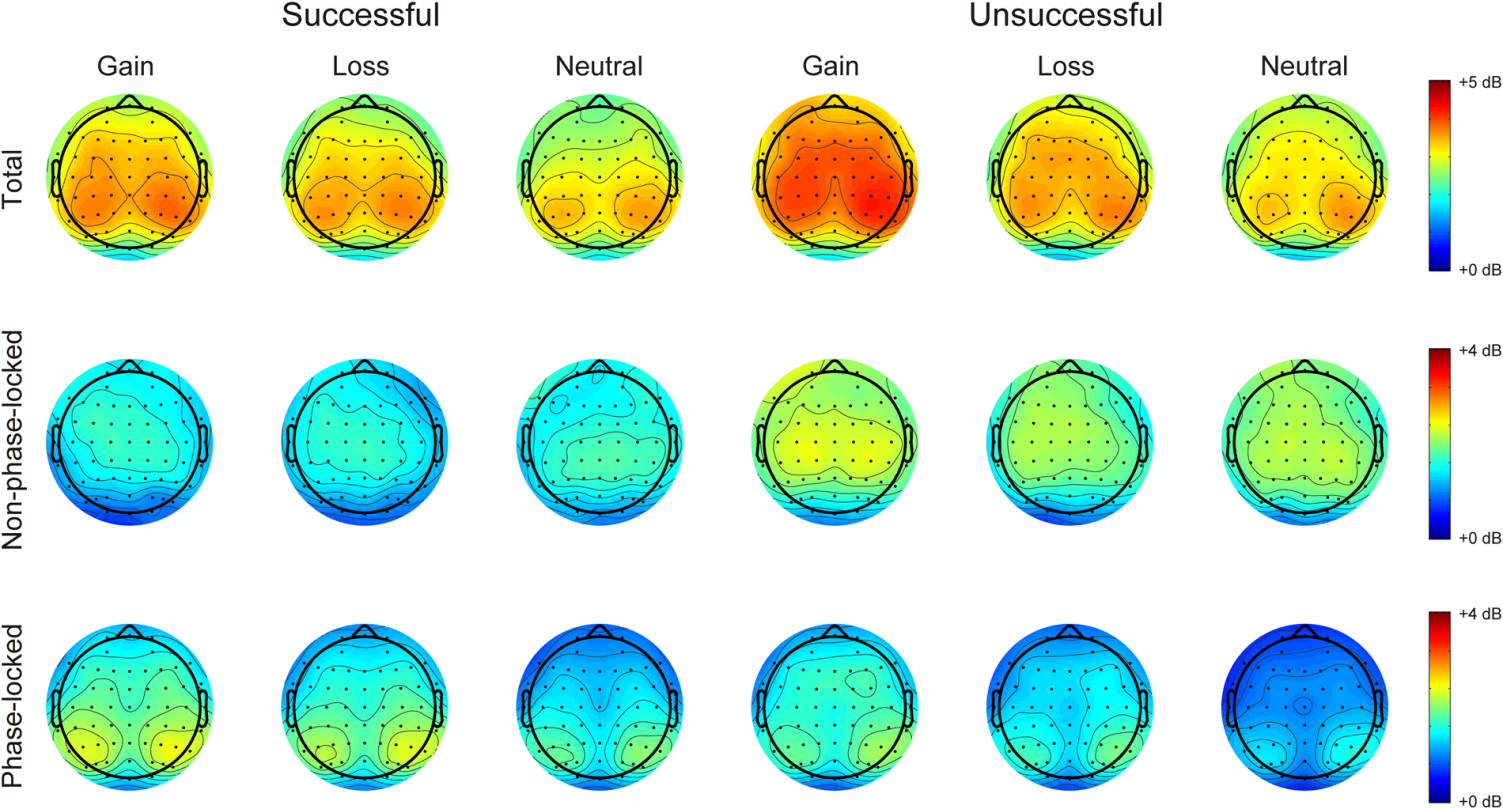
Topographic distribution maps for total, non-phase-lock, and phase-lock delta power (1–3 Hz, 200–500 ms) in response to successful and unsuccessful performance feedback as a function of context.

**Figure 7:**
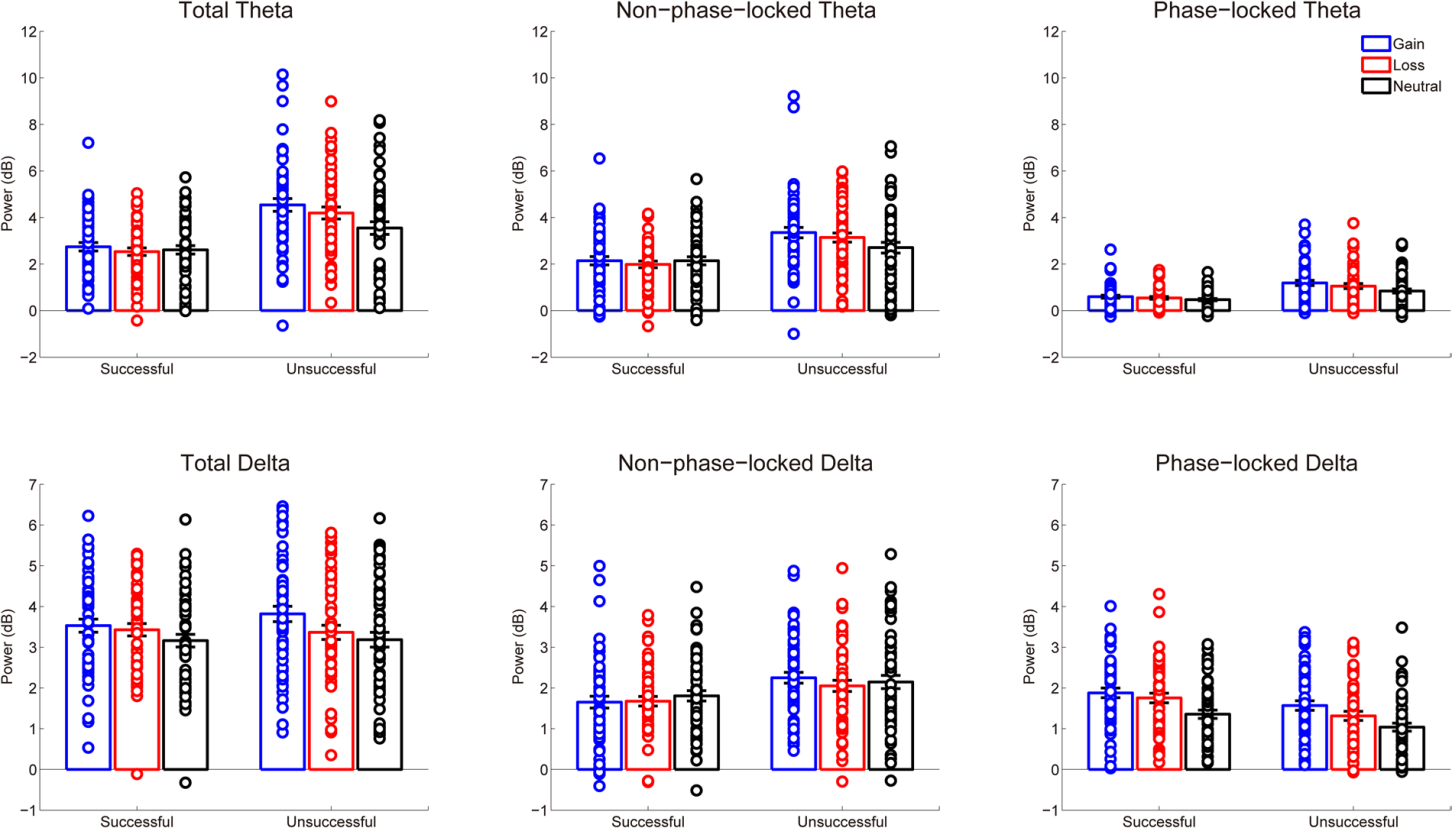
Stripcharts of power data for total, non-phase-lock, phase-lock theta and delta power in response to successful and unsuccessful performance feedback as a function of context. Error bars represent standard error of the means.

Figure 4 shows the time-frequency representations of total, non-phase-locked, and phase-locked EEG power elicited by performance feedback as a function of context. Figures 5 and 6 display the scalp distributions for theta power (4–6 Hz, 200–500 ms) and delta power (1–3 Hz, 200–500 ms), respectively. The data of theta and delta power are shown in Fig. 7. Table 3 summarizes the results of ANOVAs on total, non-phase-locked, and phase-locked theta and delta power. The ANOVA of total theta power yielded significant main effects of performance and context, which were qualified by a significant interaction between performance and context. Post hoc comparisons revealed that successful performance feedback elicited comparable total theta power across the three types of trials (*p*s > 0.7). In contrast, total theta power elicited by unsuccessful performance feedback was enhanced on both the gain (*p* < 0.001) and loss (*p* = 0.019) trials compared to the neutral trials, with no significant difference between the gain and loss trials (*p* = 0.176). The ANOVA of non-phase-locked theta power resulted in a significant main effect of performance, which was qualified by a significant interaction between performance and context. Post hoc comparisons revealed that successful performance feedback elicited comparable non-phase-locked theta power across the three types of trials (*p*s > 0.9). In contrast, non-phase-locked theta power elicited by unsuccessful performance feedback was enhanced on the gain trials as compared to the neutral trials (*p* = 0.009), with no differences between the gain and loss trials (*p* = 0.653), and between the loss and neutral trials (*p* = 0.131). With respect to phase-locked theta power, it was increased for unsuccessful relative to successful performance feedback. Context had a significant effect on phase-locked theta power. Post hoc comparisons revealed that phase-locked theta power was increased on both the gain and loss trials compared to the neutral trials (*p*s < 0.05), with no significant difference between the gain and loss trials (*p* = 0.178). Unlike non-phase-locked theta power, the interaction between performance and context failed to reach significance.

**Table 3:** Summary of the analysis of variance performed on total, non-phase-locked, and phase-locked theta and delta power.

	Total power				Non-phase-locked power				Phase-locked power			
	*df*	*F*	*p*	ω^2^	*df*	*F*	*p*	ω^2^	*df*	*F*	*p*	ω^2^
Theta												
P	(1,56)	58.73	<0.001	0.287	(1,56)	40.95	<0.001	0.183	(1,56)	38.11	<0.001	0.226
C	(2,112)	8.12	0.001	0.025	(2,112)	2.79	0.066	0.009	(2,112)	10.29	<0.001	0.033
P × C	(2,112)	6.07	0.004	0.016	(2,112)	3.88	0.025	0.012	(2,112)	2.53	0.090	0.005
Delta												
P	(1,56)	0.58	0.450	<0.001	(1,56)	21.27	<0.001	0.092	(1,56)	24.28	<0.001	0.107
C	(2,112)	14.48	<0.001	0.083	(2,112)	0.69	0.495	<0.001	(2,112)	33.53	<0.001	0.163
P × C	(2,112)	3.33	0.041	0.008	(2,112)	1.51	0.226	0.002	(2,112)	1.00	0.367	<0.001

*Note*. P = Performance; C = Context.

The ANOVA of total delta power revealed a significant main effect of context, which was qualified by a significant interaction between context and performance. Post hoc comparisons revealed that total delta power elicited by successful performance feedback on both the gain (*p* = 0.001) and loss (*p* = 0.013) trials was larger than that on the neutral trials, with no significant difference between the gain and loss trials (*p* > 0.9). In contrast, total delta power elicited by unsuccessful performance feedback was larger on the gain trials than that on both the loss (*p* = 0.001) and neutral (*p* < 0.001) trials, with no significant difference between the loss and neutral trials (*p* = 0.641). Non-phase-locked delta power was enhanced for unsuccessful compared to successful performance feedback. Both the main effect of context and the interaction effect between performance and context were not significant. With respect to phase-locked delta power, there was a significant main effect of performance such that phase-locked delta power was larger for successful than unsuccessful performance feedback. Moreover, the main effect of context was also significant. Post hoc comparisons revealed that phase-locked delta power was enhanced for the gain trials than for both the loss and neutral trials (*p*s < 0.003) and for the loss trials than for the neutral trials (*p* < 0.001). Moreover, the interaction between performance and context failed to reach significance.

## Discussion

The current study aimed to investigate whether feedback evaluation is modulated by motivational valence or by motivational salience, which remains unclear in previous studies due to methodological factors including unbalanced outcome ratios and perceptual characteristics^26–28^. This study extends beyond those previous studies to employ a similar MID task but with well-controlled experimental parameters. Moreover, to our knowledge, this is the first study to examine the oscillatory profiles of feedback evaluation under a gain context, a loss context, and a neutral context. For behavioral and rating data, we observed a response speeding and greater affective experiences that increased from the neutral context to the motivational contexts. For electrophysiological data, we obtained results supporting that feedback evaluation could be modulated either by motivational valence or by motivational salience, which was discussed in details below.

For the time-domain data, the FRN was decreased in both the gain and loss contexts as compared to the neutral context, indicating a motivational salience during the early stage (200–300 ms) of feedback evaluation. The increased FRN observed for the neutral context relative to both the gain and loss contexts is consistent with two previous studies using a similar MID task^27,28^. One possible explanation for this finding is attributable to that outcome feedback in the neutral context might be more unexpected. Specifically, participants were more engaged in the motivational (gain and loss) contexts and their outcome predictions were therefore more accurate. In contrast, participants’ responses in the neutral context were stochastic and outcome feedback was thus more unexpected. Supporting this speculation, the response times were significantly longer and the response variance was significantly larger in the neutral context than the motivational contexts. However, we acknowledged that this interpretation seems to be a little speculative since this feedback aspect (expectedness) was not assessed via a rating question, which should be addressed in future research.

**Figure 8:**
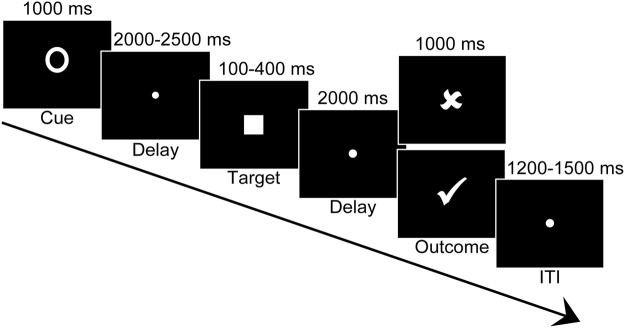
Timeline of the monetary incentive delay task. ITI = intertrial interval.

Importantly, the incentive effect of the FRN was comparable between the gain context and the loss context, suggesting that the early feedback evaluation as indexed by the FRN is driven by motivational salience. Similarly, a recent study found that the FRN was decreased as a function of incentive magnitude during both a gain context and a loss context^27^. However, our findings were at odds with the Pfabigan *et al*. study observing a larger FRN during a loss versus a gain context when participants received successful performance feedback but a reversed pattern when they received unsuccessful performance feedback. This discrepancy may be attributable to methodological differences between the two studies. In the Pfabigan *et al*. study, successful feedback was “+2€” in the gain context and “0€” in the loss context, whereas unsuccessful feedback was “0€” in the gain context and “−2€” in the loss context. Therefore, the findings in the previous study appeared to be confounded with visual characteristics^30^. In contrast, in the current study, successful performance feedback was delivered with the same tick and unsuccessful performance feedback by the same cross across contexts (Fig. 8), making feedback stimuli more comparable between contexts. Another explanation concerns task differences. An influential study has demonstrated that the FRN encodes whatever feedback dimension is most salient in the investigated task^15^. Specifically, when feedback stimuli convey both utilitarian and performance information, the FRN is sensitive to both aspects of the feedback, depending on which aspect is more emphasized. Whereas the Pfabigan *et al*. study emphasized the utilitarian feedback aspect, the current MID task version emphasized the performance feedback aspect. This might explain why two main effects were observed in the current study, while an interaction effect was obtained in the previous study^28^. These inconsistent results across the studies highlight that one should keep in mind that the FRN is very susceptible to methodological issues when choosing the task set-up^12^.

In contrast to the FRN, which represents the fast, coarse evaluation of feedback stimuli, the P3 is suggested to index the allocation of attentional resources based on motivational significance during decision making^41^. From this perspective, it might be expected that modulation of context manipulation would be more obvious during the late stage of feedback evaluation as indexed by the P3. Indeed, we observed a reward valence effect during this stage such that both the posterior P3 and the anterior P3 were larger during the gain context than during the loss context, which was in turn greater than during the neutral context. Moreover, this reward priority in allocation of attentional resources remained regardless of whether participants received successful or unsuccessful performance feedback. It is unsurprising that the P3 elicited by successful performance feedback was larger in the gain relative to loss context, because a successful response would result in a winning of Ұ1 in the gain context but Ұ0 in the loss context. Interestingly, the P3 elicited by unsuccessful feedback was also larger in the gain context (resulting in Ұ0) compared to the loss context (resulting in a loss of Ұ1). We admit that it is unclear why the lower objective-magnitude outcome (Ұ0 in the gain context) elicited a larger P3 than the higher objective-magnitude outcome (Ұ1 in the loss context), but it might reflect that outcomes in the gain context were more target-related and thus were imbued with greater task relevance^42^. Future research is needed to address this issue more directly.

Since ERP components, especially the FRN, is highly susceptible to component overlap^33^, we therefore extracted the time-frequency data of theta and delta power during feedback evaluation. We found that phase-locked theta-band activity over the midfrontal cortex showed similar patterns as the FRN. Specifically, phase-locked theta power was increased during both the gain and loss contexts relative to the neutral context with no significant difference between the gain and loss contexts, thus indicating that phase-locked theta power is mainly driven by motivational salience instead of motivational valence^37^. According to an influential theory^43^, theta power has been proposed to reflect the recruitment of cognitive control, because it is sensitive to various situations that need to exert cognitive control, such as error commission, conflict, novelty, and as in the present study, motivational salience. Therefore, our finding of enhanced theta power in the motivational versus neutral context appears to reflect a need to increase cognitive control when participants had a chance to obtain rewards in the gain context or to avoid punishments in the loss context. Unlike phase-locked theta power, non-phase-locked theta power was comparable across contexts when participants made a successful response. However, non-phase-locked theta power was enhanced during the gain context compared to the neutral context when participants made an unsuccessful response, indicating a reward valence effect only for unsuccessful feedback. The reward valence effect observed for non-phase-locked theta power was in contrast to the motivational salience effect observed for phase-locked theta power. A recent theory suggests that non-phase-locked and phase-locked components reflect different cognitive processes, with phase-locked power indexing bottom-up neural activity and non-phase-locked power indexing top-down modulation^44^. According to this theory, it is possible that the reward valence effect is driven by top-down brain activity, because monetary incentive in this task was less salient relative to performance feedback and thus needed more elaborative processing in the brain. In line with this explanation, a previous study has demonstrated that the conflict-related theta power contains non-phase-locked, rather than phase-locked, oscillations^45^.

With respect to delta power, its phase-locked component was enhanced for successful relative to unsuccessful performance feedback, which is consistent with recent studies^34,36,38^. Moreover, phase-locked delta power showed a reward valence effect such that it was linearly increased from the neutral context, to the loss context, and to the gain context, which is in contrast to the fact that the phase-locked theta power was comparable between the gain and loss contexts. Unlike theta power, the functional significance of delta power in incentive processing remains unclear since it has not been received much attention until recently^34–36^. A previous research has demonstrated that whereas theta power reflects the most salient information in the task context, delta power reflects more elaborative processing including both primary and secondary feedback properties^34^. In our experiment, performance feedback constituted the primary information whereas monetary incentive was the secondary information. Our delta findings are therefore consistent with the view that delta power indexes more elaborative processing such that the behavioral and rating patterns were mirrored by delta power instead of theta power. However, the functional significance of delta power should be clarified in future research.

Some findings in our study can be couched with the framework of reinforcement learning theory. Our performance main effects (as revealed by the FRN, the anterior P3, the theta power, and the delta power) across contexts primarily reflect reinforcement versus punishment as described by the learning theory^5^, which were the strongest effects in terms of effect size. In a learning theory sense, successful performance feedback is referred to as reinforcement, which consists of positive reinforcement (i.e., the presentation of reward: a winning of Ұ1 in the gain context) and negative reinforcement (i.e., the removal of an aversive stimulus: Ұ0 in the loss context). In contrast, unsuccessful performance feedback is referred to as punishment, which consists of the presentation of an aversive stimulus (a loss of Ұ1 in the loss context) and the removal of an appetitive stimulus (Ұ0 in the gain context). Similarly, successful and unsuccessful performance feedback in the neutral context carry the properties of reinforcement and punishment, respectively, but with weaker intensity. Seen from this background, motivational salience would simply be expected to amplify reinforcement learning effects because both reinforcement and punishment would be expected to amplify effects as compared to a neutral control condition. That is what the interaction effects for total theta and total delta power might indicate: amplification of the main performance effect for the motivational (gain and loss) versus non-motivational (neutral) context. However, it should be noted that one potential limitation in the current study is that performance feedback in the neutral condition might not be completely neutral, which might be associated with high-level affective evaluations such as pride (successful performance) and shame (unsuccessful performance). This may be the reason why performance effects were strong overall in the study even in the neutral context.

Until now, three previous EEG studies have used a very similar MID task including a gain context, a loss context, and a neutral context, as did in the current experiment^26–28^. Our study differed from these studies, however, in two important ways. First, whereas the results of the previous studies might be confounded with either outcome probability^26,27^ or visual characteristic^27,28^, the two critical factors, which constitute two methodological considerations in recent EEG study of incentive processing^12^, have been carefully controlled in the current study such that outcome probability was adjusted at about 0.5 and visual characteristics of outcome stimuli were same across contexts. Second, the previous studies have focused on ERP responses to feedback evaluation but not the synchronization of EEG frequency bands (theta and delta). The current study is the first one that investigated the neural signals of feedback evaluation during both the time domain and the time-frequency domain.

In sum, the fact that EEG signals elicited by feedback stimuli are sensitive to either performance feedback^3^ or motivational feedback^6^ indicates an interface between cognitive and affective processes associated with feedback evaluation^46^. Overall, our data are consistent with previous research demonstrating that motivational and performance feedback modulate these EEG signals in an additive way^23,24,37^, but go a further step to demonstrate that this affective modulation on the feedback evaluation can be driven either by motivational valence or by motivational salience. The sensitivity of feedback evaluation to the valence and salience aspects of the feedback depends on the assessment methods as diverse as temporal dynamics (the FRN vs. the P3), frequency dynamics (theta vs. delta power), and phase dynamics (evoked vs. induced power).

## Methods

The data reported in this study are from the non-overlapping results of an MID task in a previous study^40^. Specifically, whereas the previous study focused on EEG signals during the anticipatory phase of the MID task and their relationship with a behavioral task, the present study analyzed EEG signals during the feedback phase of the MID task. Also note that one participant in the current study was not included in the previous study, due to his interruption on the behavioral task.

### Participants

Fifty-seven right-handed undergraduates (30 females and 27 males, 17–23 years of age) participated in this study. All participants had normal or corrected-to-normal visual acuity and were free of neurological and psychological disorders. Written informed consent was obtained from all participants and parents of two participants under the age of 18 years before the experiment. Each participant received a base payment of Ұ30 (roughly equals to $4.50) for participation, plus a bonus money (about Ұ30) based on their performance in the experimental session. This study was approved by the Dalian Medical University Institutional Review Board. All methods were carried out in accordance with the relevant guidelines and regulations.

### Procedure

In the MID task (Fig. 8), participants on each trial saw one of three cues (1000 ms) indicating a potential monetary gain (a plus sign), a potential monetary loss (a minus sign), or no money at stake (an empty circle). After a jittered delay (2000–2500 ms), participants pressed a response button as fast as possible to a visual target (a white square). Target duration of each condition was initially set to 250 ms and then was adjusted between 100 and 400 ms via a staircase procedure whereby it was decreased or increased by 25 ms following a successful or unsuccessful response, respectively. This procedure resulted in an averaged target duration of 232 ms and an approximately 50% success rate for each condition. After a second delay (2000 ms), participants saw the performance feedback (1000 ms) of their response on that trial, with a successful response indicated by a white tick and an unsuccessful response by a white cross. The tick feedback on gain trials indicated a winning of Ұ1 whereas the cross feedback in loss trials indicated a loss of Ұ1. Other feedback (i.e., the cross on gain trials, the tick on loss trials, the cross and tick on neutral trials) indicated that participants neither gained nor lost any money. Each trial ended with an intertrial interval (1200–1500 ms).

The task consisted of 240 trials, which were divided into four blocks (20 trials for each cue in each block) with a break between blocks. Participants were endowed with an initial bankroll of Ұ30 and were told that they should respond as quickly as possible to the visual target regardless of the type of the cue. Participants played 18 training trials prior to the experiment. After blocks 2 and 4, participants rated their affective responses during the anticipation and receipt of outcomes under all incentive conditions via a 5-point scale in terms of valence (1 = negative and 5 = positive) and arousal (1 = low intensity and 5 = high intensity).

### EEG recording

Continuous EEG was recorded using an electrode cap with 64 Ag/AgCl electrodes mounted according to the extended International 10/20 system. The signals were recorded using a left mastoid reference electrode and rereferenced offline to the mean of the activity at the left and right mastoids. Horizontal and vertical electrooculogram (EOG) were recorded with two pairs of electrodes. The EEG and EOG were amplified using a Neuroscan SynAmps^2^ amplifier with a low-pass of 100 Hz in DC acquisition model and digitalized at a sample rate of 500 Hz. Electrode impedance was kept under 5 KΩ throughout the experiment. The EEG data were then analyzed in MATLAB 2014a using EEGLAB toolbox (v13.1.1)^47^.

### Time-domain analyses

The EEG data were linearly detrended, filtered with a band-pass of 0.1 and 30 Hz (roll-off 6 dB/octave), and then epoched from −1000 to 1500 ms relative to feedback onset with the activity from −200 to 0 ms serving as the baseline. All epoched data were screened manually for artifacts (e.g., spikes, drifts, and nonbiological signals) and then were subjected to an infomax independent component analysis (runica)^47,48^. Individual components were inspected and blink components were removed. Additional artifacts were removed using a semi-automated procedure with the following criteria: a step more than 50 μV between sample points, a voltage difference exceeding 200 μV within a trial, or a maximum voltage difference less than 0.5 μV within 100-ms intervals^4^. Artifact-free epochs were averaged separately for each feedback condition. Preliminary analysis on the number of the accepted ERP trials revealed no condition effects (*p*s > 0.05): the gain context (successful feedback: *M* = 37.30, *SD* = 3.36; unsuccessful feedback: *M* = 35.81, *SD* = 3.48), the loss context (successful feedback: *M* = 37.04, *SD* = 3.47; unsuccessful feedback: *M* = 35.05, *SD* = 4.46); the neutral context: (successful feedback: *M* = 38.19, *SD* = 2.53; unsuccessful feedback: *M* = 34.44, *SD* = 5.04).

**Figure 9:**
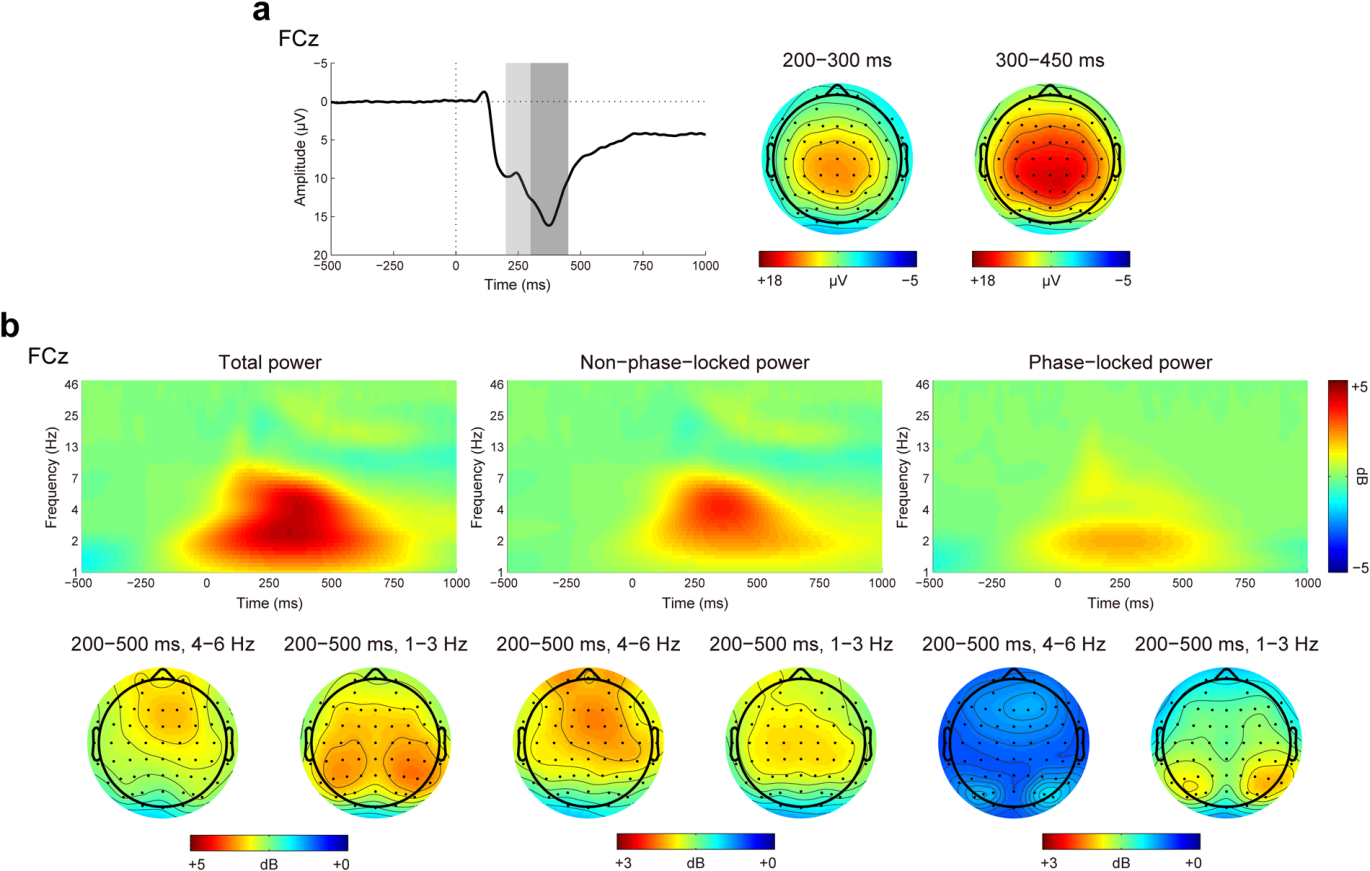
Time domain and time-frequency domain data associated with FCz, averaged over all conditions. (**a**) Grand-averaged ERP waveforms and scalp distributions for the FRN and P3. The shaded areas depict the time windows during which the FRN (200–300 ms), and P3 (300–450 ms) were scored. (**b**) Time-frequency representations of EEG powers and scalp distributions for theta power and delta power (from left to right): total power, non-phase-locked power, and phase-locked power.

ERP components were scored as the mean voltage of different time windows at representative electrodes: the FRN from 200 to 300 ms at FCz and the P3 from 300 to 450 ms at CPz. The selection of time windows and electrodes was based on ERP waveforms and topographic maps collapsed across conditions (Fig. 9a), which thus was orthogonal to the conditions of interest^49^. To corroborate our FRN findings obtained using the mean-amplitude approach, the FRN was quantified using a mean-to-mean-amplitude approach, that is, the difference between the mean values in 20 ms windows surrounding the positive peak (P2: 196 ms) and the negative peak (FRN: 246 ms) at FCz (calculated as FRN-P2). This mean-to-mean-amplitude approach accounts in part for the component overlap between the FRN and the P2. In addition, visual inspection of the ERP waveforms (Fig. 9a) indicated a large P3 (P3a) at FCz following the FRN, and this anterior P3 was scored with the same parameters as the P3 at CPz (the posterior P3).

### Time-frequency decomposition

Time-frequency decomposition was performed using custom-written MATLAB routines to isolate theta and delta power. The processing stream was similar to the time-domain analyses, except that no filtration was applied and a wider epoch (−4000 ms to 3000 ms) was created to allow for the discarding of edge effects. After removing artifacts described earlier, time-frequency decomposition was performed by multiplying the fast Fourier transformed (FFT) power spectrum of single trial EEG data with the FFT power spectrum of a set of complex Morlet wavelets that were defined as a Gaussian-windowed complex sine wave: *e*^*i*2π*tf*^${e}^{-{t}^{2}/(2{\sigma }^{2})}$, where the *t* is time, *f* is frequency, which increased from 1 to 50 Hz in 50 logarithmically spaced steps, *σ* defines the width of each frequency band, set according to 4/(2π*f*), and taking the inverse FFT. The end result of this process is identical to time-domain signal convolution and provides estimates of instantaneous power (the magnitude of the analytic signal), defined as *z*[*t*] (power time series: *p*(*t*) = real[*z*(*t*)]^2^ + imag[*z*(*t*)]^2^). Each epoch was then cut in length (−500 to 1000 ms) to account for edge effects and power was normalized by conversion to a decibel (dB) scale (10*log10[power(*t*)/power(baseline)]) from a baseline of 300–200 ms prior to feedback onset, allowing a direct comparison of effects across frequency bands.

Because EEG power within a given frequency band consists of a phase-lock (evoked) component that gives rise to the ERP and a non-phase-locked (induced) component that cannot be attributed to the ERP^44^, the total delta and theta power was decomposed into phase-locked and non-phase-locked components according to previous studies^45^. The non-phased-locked power was obtained by subtracting the ERP for each condition from the time-domain EEG signals on each trial and then performing the time-frequency decomposition as described above. The phase-locked power was computed by subtracting the non-phase-locked power from the total power. Similar as ERP components, the measurement parameters for total power, non-phase-locked power, and phase-locked power were defined based on the time-frequency representations and scalp distributions of total power averaged over all conditions (Fig. 9b). Specifically, theta power was scored as the mean activity from 200 to 500 ms over 4–6 Hz at FCz, whereas delta power from 200 to 500 ms over 1–3 Hz at CPz. These parameters are similar to previous research on feedback-locked theta and delta power^36,37^.

Scores from the time domain and time-frequency domain were evaluated statistically using SPSS (v20.0). All data were analyzed with repeated measures analyses of variance (ANOVAs) with Context (gain vs. loss vs. neutral) and Performance (successful vs. unsuccessful) as within-subjects factors. Greenhouse-Geisser epsilon correction was applied when factors had more than two levels and Bonferroni correction was used for post hoc comparisons.

### Data availability

The datasets are available upon reasonable request.
